# Intranasal Boosting with Spike Fc-RBD of Wild-Type SARS-CoV-2 Induces Neutralizing Antibodies against Omicron Subvariants and Reduces Viral Load in the Nasal Turbinate of Mice

**DOI:** 10.3390/v15030687

**Published:** 2023-03-06

**Authors:** Jian-Piao Cai, Cuiting Luo, Kun Wang, Hehe Cao, Lin-Lei Chen, Xiaojuan Zhang, Yuting Han, Feifei Yin, Anna Jinxia Zhang, Hin Chu, Shuofeng Yuan, Kin-Hang Kok, Kelvin Kai-Wang To, Honglin Chen, Zhiwei Chen, Dong-Yan Jin, Kwok-Yung Yuen, Jasper Fuk-Woo Chan

**Affiliations:** 1 State Key Laboratory of Emerging Infectious Diseases, Carol Yu Centre for Infection, Department of Microbiology, School of Clinical Medicine, Li Ka Shing Faculty of Medicine, The University of Hong Kong, Pokfulam, Hong Kong SAR, China; 2 Key Laboratory of Tropical Translational Medicine of Ministry of Education, Hainan Medical University, Haikou 570100, China; 3 Academician Workstation of Hainan Province, Hainan Medical University-The University of Hong Kong Joint Laboratory of Tropical Infectious Diseases, Hainan Medical University, Haikou 570100, China; 4 Centre for Virology, Vaccinology and Therapeutics, Hong Kong Science and Technology Park, Hong Kong SAR, China; 5 Guangzhou Laboratory, Guangdong Province, Guangzhou 510000, China; 6 School of Biomedical Sciences, Li Ka Shing Faculty of Medicine, The University of Hong Kong, Pokfulam, Hong Kong SAR, China

**Keywords:** COVID-19, SARS-CoV-2, Omicron variant, BA.5.2, XBB.1, Fc-RBD, CoronaVac, neutralizing antibody

## Abstract

The emergence of new immune-evasive severe acute respiratory syndrome coronavirus 2 (SARS-CoV-2) variants and subvariants outpaces the development of vaccines specific against the dominant circulating strains. In terms of the only accepted immune correlate of protection, the inactivated whole-virion vaccine using wild-type SARS-CoV-2 spike induces a much lower serum neutralizing antibody titre against the Omicron subvariants. Since the inactivated vaccine given intramuscularly is one of the most commonly used coronavirus disease 2019 (COVID-19) vaccines in developing regions, we tested the hypothesis that intranasal boosting after intramuscular priming would provide a broader level of protection. Here, we showed that one or two intranasal boosts with the Fc-linked trimeric spike receptor-binding domain from wild-type SARS-CoV-2 can induce significantly higher serum neutralizing antibodies against wild-type SARS-CoV-2 and the Omicron subvariants, including BA.5.2 and XBB.1, with a lower titre in the bronchoalveolar lavage of vaccinated Balb/c mice than vaccination with four intramuscular doses of inactivated whole virion vaccine. The intranasally vaccinated K18-hACE2-transgenic mice also had a significantly lower nasal turbinate viral load, suggesting a better protection of the upper airway, which is the predilected site of infection by Omicron subvariants. This intramuscular priming and intranasal boosting approach that achieves broader cross-protection against Omicron variants and subvariants may lengthen the interval required for changing the vaccine immunogen from months to years.

## 1. Introduction

The coronavirus disease 2019 (COVID-19) pandemic caused by the severe acute respiratory syndrome coronavirus 2 (SARS-CoV-2) has infected over 600 million people and caused over 6 million deaths after 3 years [1]. While social distancing, universal masking, screening by rapid diagnostic tests, isolation of test-positive cases, quarantine of contacts, and early treatment of cases with risk factors by antivirals or neutralizing antibodies are the key control measures in the early phase of the pandemic, vaccination has become critical for converting this pandemic disease into an endemic disease [2,3,4]. Although the presently available COVID-19 vaccines mainly prevent severe disease and cannot completely stop infection, the hybrid immunity generated by these vaccines together with natural infection may be sufficient for the vaccinated population to live at pre-pandemic normalcy [5]. The scientific community, pharmaceutical industry, and government regulatory agencies deserve credit for being able to push COVID-19 vaccines into clinics within 12 months. These vaccines use the SARS-CoV-2 spike containing the receptor binding domain (RBD) of the wild-type SARS-CoV-2 to induce high titers of neutralizing antibodies—the most important correlate of vaccine protection in field studies [6,7,8]. However, SARS-CoV-2 can mutate and recombine to form new variants and subvariants of concern that can often replace the preceding dominant strains within months [9,10]. The most recent Omicron subvariants, such as the recombinant XBB.1 and BA.5, can largely evade the neutralizing antibodies induced by natural infection or vaccines against the wild-type SARS-CoV-2 [11,12,13,14]. It would be important to find a vaccine that can generate broad-spectrum neutralizing antibodies so that repeated immunizations with the same vaccine can continue to offer protection against new variants and subvariants without the need to change the immunogen of the vaccine [15]. Any change in the vaccine immunogen may arouse anxiety from the public and require a lengthy and expensive process from preclinical investigations to the three phases of clinical trials before emergency approval. In this mouse vaccination study, we showed that repeated intranasal boosting by recombinant Fc-linked spike RBD after two doses of priming with inactivated vaccine is already sufficient to induce modest levels of antibodies against the Omicron subvariants, which cannot be achieved by an equal number of boosting with the original inactivated vaccine. The findings and implications of this approach are analyzed and discussed.

## 2. Materials and Methods

### 2.1. Viruses and Biosafety

The wild-type SARS-CoV-2 strain HKU-001a (GenBank: MT230904) was a clinical isolate as previously described [16]. The SARS-CoV-2 BA.5.2 isolate (GISAID accession number EPI_ISL_13777658) and XBB.1 isolate (GISAID accession number EPI_ISL_15602393) were isolated from laboratory-confirmed COVID-19 patients in Hong Kong [14]. In vitro and in vivo experiments involving infectious wild-type SARS-CoV-2 and Omicron subvariants were performed in a biosafety level 3 laboratory at the Department of Microbiology, The University of Hong Kong, and strictly followed the approved standard operating procedures [17].

### 2.2. Cell Lines

ExpiSf9 cells [Thermo Fisher Scientific Inc.; Cat# A35243] and Sf9 cells [Thermo Fisher Scientific Inc.; Cat# 11496015] were used to generate recombinant proteins. VeroE6/TMPRSS2 cells (JCRB cell bank of Okayama University; Cat# JCRB1819) were used for conventional live virus neutralization tests (cVNT). All cell lines used in this study were routinely tested for mycoplasma contamination and found to be mycoplasma-free.

### 2.3. Expression and Purification of RBD, Fc-RBD, Subunit 1, and Full-Length Spike of SARS-CoV-2

**Figure 1:**
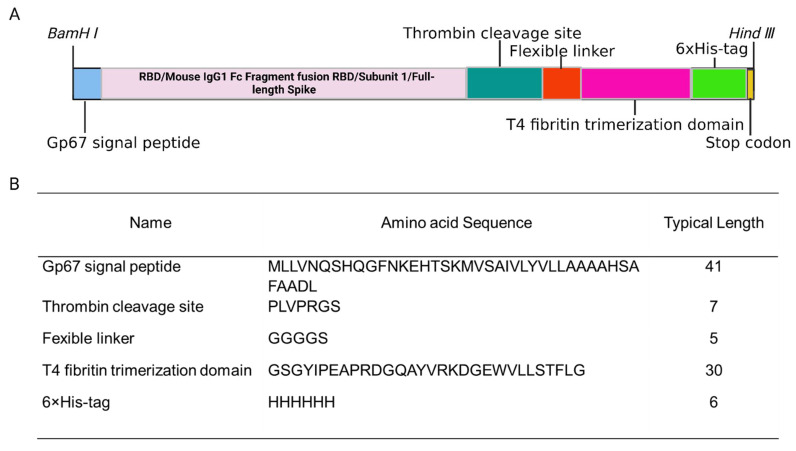
Design of booster vaccines in this study. (**A**) A schematic showing the gene constructs used for recombinant protein expression. (**B**) Amino acid sequences in the recombinant protein expression construction. The figure was prepared with BioRender (https://biorender.com; accessed on 22 January 2023).

Recombinant receptor-binding domain (RBD) (residues 306F-543F) of SARS-CoV-2 spike protein from the reference sequence Wuhan-Hu-1 (GenBank ID YP_009724390.1) (wild-type) or mouse IgG1 Fc fragment fusion RBD (Fc-RBD), subunit 1 (22T-682R) (S1), and full-length spike (S) were expressed and purified in insect cells as previously described with modifications [18] (Figure 1). Briefly, gene sequences were baculovirus-codon-optimized and cloned into the pFast dual baculovirus expression vector (Sangon Biotech, Shanghai, China). The constructs were fused with an N-terminal gp67 signal peptide and a C-terminal 6xHis tag for secretion and purification. The C-terminal T4 fibritin trimerization motif, a flexible linker, and a thrombin cleavage site were included for trimeric folding. To express the Fc-RBD, residues 306F-543F of SARS-CoV-2 (the Wuhan-Hu-1 strain) were cloned downstream of a monomeric mouse Fc tag. Lastly, recombinant bacmid DNA was generated using the Bac-to-Bac system (Thermo Fisher Scientific, Waltham, MA, USA; Cat# 10359016). Baculovirus was produced by transfecting purified bacmid DNA into Sf9 cells using Cellfectin (Thermo Fisher Scientific, Waltham, MA, USA Cat# 10362100) and subsequently used to infect ExpiSf9 cell suspension culture (Thermo Fisher Scientific, Waltham, MA, USA, Cat# A35243) at a multiplicity of infection of 1–10. Infected ExpiSf9 cells were incubated at 27.5 °C with shaking at 125 r.p.m. for 96 h for protein expression. The supernatant was collected and then concentrated using a 10 kDa MW or 50 kDa MW cutoff lab-scale TFF system (Millipore, Burlington, MA, USA). The protein was purified by a Ni-NTA purification system (Thermo Fisher Scientific, Waltham, MA, USA, Cat#: R90110), followed by size exclusion chromatography (Bio-Rad, Hercules, CA, USA, ENrich^TM^ SEC 650 10 × 300 Column Cat#: 7801650), and buffer exchanged into phosphate buffered saline (PBS), pH 7.4. The purified protein concentration was determined using the Bradford Assay Kit (Bio-Rad, Hercules, CA, USA, Cat#: 5000002) according to the manufacturer’s instructions. The purity of recombinant proteins was verified using sodium dodecyl sulfate-polyacrylamide gel electrophoresis (SDS-PAGE).

### 2.4. Animals

The animal experiments were approved by the Committee on the Use of Live Animals in Teaching and Research (CULATR) of The University of Hong Kong. Heterogenous K18-hACE2 transgenic mice [2B6.Cg-Tg (K18-ACE2)2Prlmn/J] were obtained from the Jackson Laboratory [19].

### 2.5. Vaccination Procedure

**Figure 2:**
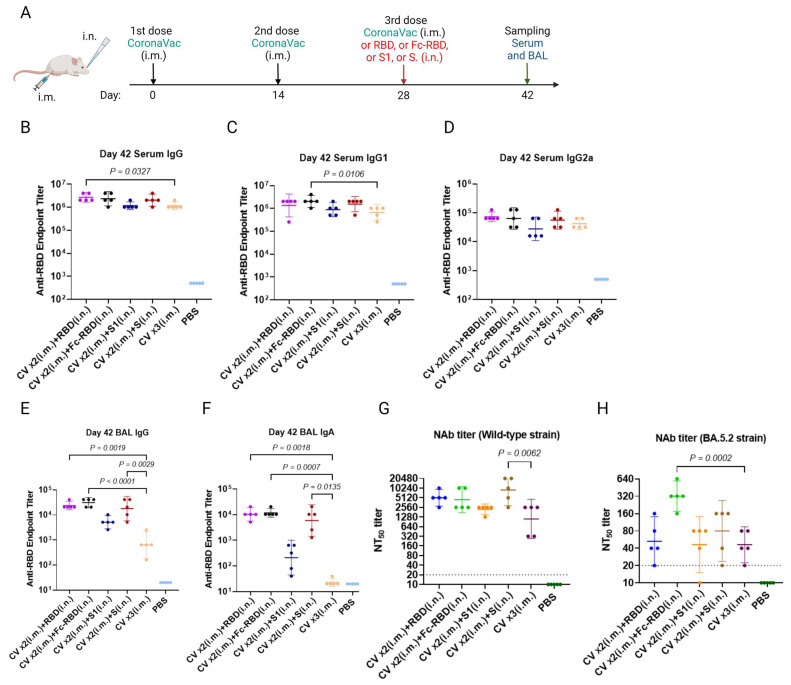
Immune responses after two doses of intramuscular CoronaVac followed by one dose of intranasal vaccine booster. (**A**) A schedule of Balb/c mouse vaccination and sample collection. Vaccinated mouse serum anti-SARS-CoV-2 wild-type spike RBD antibodies on day 42 were detected by EIA and presented as (**B**) IgG; (**C**) IgG1; and (**D**) IgG2a endpoint titers. Bronchoalveolar lavage (BAL) anti-SARS-CoV-2 spike RBD (**E**) IgG and (**F**) IgA on day 42 were determined by EIA. (**G**) Wild-type SARS-CoV-2 and (**H**) Omicron BA.5.2 subvariant neutralization of pooled serum samples collected on day 42 was quantitated by conventional live virus neutralization tests (cVNT). A one-way ANOVA test was used for statistical analysis. Abbreviations: BAL, Bronchoalveolar lavage; CV, CoronaVac; i.m., intramuscular; and i.n., intranasal.

**Figure 3:**
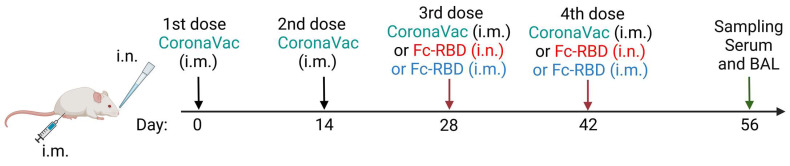
Comparison of humoral immune responses between two intramuscular doses of CoronaVac plus two intranasal Fc-RBD or two doses of intramuscular Fc-vaccine booster with four intramuscular doses of CoronaVac in Balb/c mice. Schedule of Balb/c mouse vaccination and sample collection. Abbreviations: i.m., intramuscular; i.n., intranasal; and BAL, Bronchoalveolar lavage.

Balb/c mice vaccination. Four–six-week-old female Balb/c mice were randomly divided into six groups (n = 5 per group). In the first four groups, each mouse was vaccinated with two doses of CoronaVac (Sinovac) (100 µL per mouse) intramuscularly on day 0 and day 14. An intranasal booster (trimeric RBD, trimeric Fc-RBD, trimeric S1, or trimeric S) (25 µg in 20 µL 1 × PBS) was administered intranasally to each mouse on day 28. Each mouse in the other two groups received three doses of either an equal volume of 1 × PBS as negative control or CoronaVac as the positive control on day 0, day 14, and day 28. On day 42, sera and bronchoalveolar lavage (BAL) fluids were collected for the detection of RBD-specific antibodies and neutralizing antibodies against wild-type SARS-CoV-2 and variants (Figure 2). In another set of experiments, each mouse received either four doses of CoronaVac (50 µL per mouse) or two doses of CoronaVac (50 µL per mouse) plus two doses of trimeric Fc-RBD vaccination via intranasal or intramuscular routes (Figure 3). Sera and BAL were collected on day 56.

**Figure 4:**
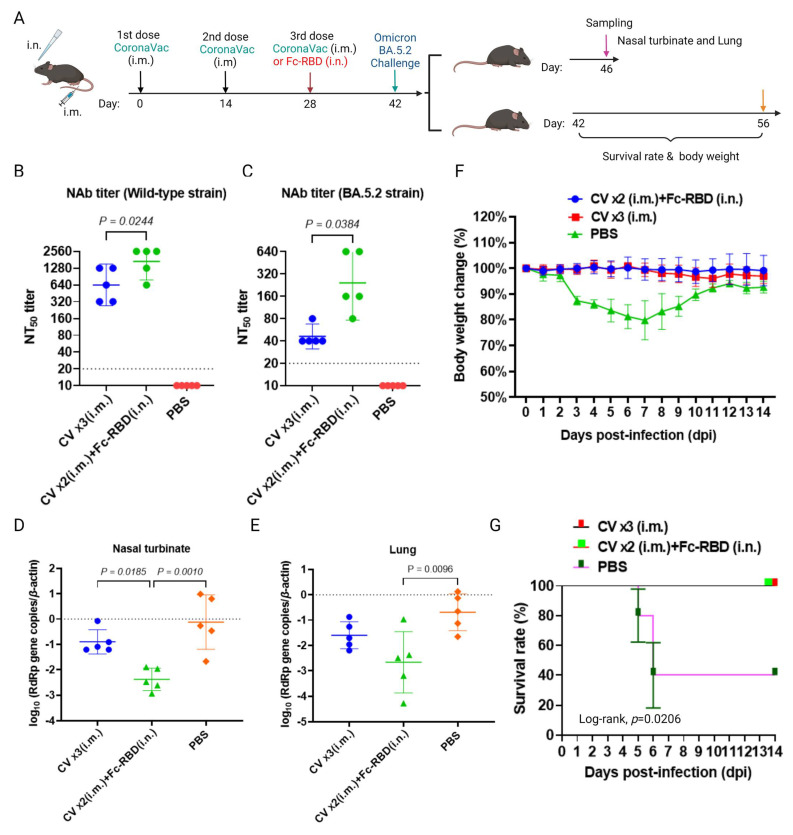
Comparison of the immunogenicity of three intramuscular doses of CoronaVac with two intramuscular doses of CoronaVacs plus one dose of intranasal vaccine booster in the K18-hACE2 mouse model. (**A**) A schedule of K18-hACE2 mouse vaccination and the SARS-CoV-2 Omicron BA.5.2 subvariant challenge. (**B**) Wild-type SARS-CoV-2; and (**C**) Omicron BA.5.2 subvariant neutralization of pooled sera collected on day 42 was quantitated by conventional live virus neutralization tests (cVNT). The viral loads of SARS-CoV-2-challenged K18-hACE2 mice were detected in the (**D**) nasal turbinates and (**E**) lung tissues. (**F**) The body weight of SARS-CoV-2-challenged K18-hACE2 mice was monitored to evaluate the effectiveness of vaccination. (**G**) The survival rates of SARS-CoV-2-challenged K18-hACE2 mice were determined using the Kaplan–Meier method, and the resulting survival curves were compared using the log-rank test. A one-way ANOVA test was used for statistical analysis. Abbreviations: CV, CoronaVac; i.m., intramuscular; and i.n., intranasal.

K18-hACE2 mice vaccination. Four–eight weeks old female and male K18-hACE2 mice were randomly divided into four groups (n = 10 per group). In group 1, each mouse was vaccinated with three doses of CoronaVac intramuscularly (50 µL per mouse) on day 0, day 14, and day 28. In group 2, each mouse was vaccinated with two doses of CoronaVac on day 0 and day 14, followed by an intranasal booster containing trimeric Fc-RBD (25 µg in 25 µL 1 × PBS) on day 28. In group 3, each mouse received an equal volume of 1 × PBS as the negative control at the same timepoints. On day 42, sera were collected for a neutralizing antibody assay against wild-type SARS-CoV-2 and Omicron subvariants (Figure 4).

### 2.6. SARS-CoV-2 Omicron BA.5.2 Variant Challenge

The K18-hACE2 mice were anesthetized with ketamine and xylazine as previously described, followed by intranasal inoculation with 20 μL of BA.5.2 1 × 10^5^ PFU per mouse as previously described [20,21]. On day 4 post-virus challenge, five mice in each group were sacrificed. The nasal turbinates and lung were collected for virological analyses. The remaining mice were kept for body weight monitoring for another 14 days and sacrificed on day 56 (Figure 4).

### 2.7. Detection of Viral Antigen-Specific IgG, IgG1, IgG2a, and IgA in Mouse Serum and/or BAL Samples by Enzyme Immunoassay (EIA)

Purified wild-type SARS-CoV-2 spike RBD was used for EIA as previously described with modifications [22]. Each well of the 96-well immunoplates (Nunc Immuno modules; Nunc, Denmark Nunc, Denmark, Cat# 1690) was coated with 50 ng of recombinant RBD protein in 50 µL 0.05 M NaHCO_3_ (pH 9.6). The plate was incubated at 4 °C overnight and then blocked with blocking reagent at 37 °C for 2 h. Heat-inactivated serum samples were serially diluted at 2-fold starting at 1:500 dilution, and heat-inactivated BAL samples were serially diluted at 2-fold starting at 1:20 dilution. A total of 50 µL of diluted serum, or BAL, was added to the wells and incubated at 37 °C for 1 h. The attached mouse IgG, IgG1, IgG2a, and IgA were detected using horseradish peroxidase (HRP)-conjugated goat anti-mouse IgG antibodies (Thermo Fisher Scientific, Waltham, MA, USA, Cat# 31430), goat anti-mouse IgG1 antibodies (Thermo Fisher Scientific, Waltham, MA, USA, Cat# PA174421), goat anti-mouse IgG2a antibodies (Thermo Fisher Scientific, Waltham, MA, USA, Cat# M32207), and goat anti-mouse IgA antibodies (Thermo Fisher Scientific, Waltham, MA, USA, Cat# PA174397), respectively. The reactions were developed by adding diluted 3,3′,5,5′-tetramethylbenzidine single solution (Invitrogen, Thermo Fisher Scientific, Waltham, MA, USA, Cat# 002023) and stopped with 0.3 N H_2_SO_4_. The optical density (OD) was read at 450 nm and 620 nm. To determine the cut-off value for positivity, mock control serum samples from unvaccinated naïve mice were used as a negative control for setting the EIA OD cut-off value. The cut-off OD was set at the mean OD of naïve mouse serum samples at all dilutions plus 3 standard deviations. The serum dilutions yielding an OD above the cut-off value were calculated.

### 2.8. Viral Culture

Viral culture was performed as previously described [16]. Briefly, TMPRSS2-expressing VeroE6 (VeroE6/TMPRSS2) cells were seeded with 10 mL of Dulbecco’s minimum essential medium (DMEM) (Gibco^®^, Thermo Fisher Scientific, Waltham, MA, USA, Cat# 11965092) containing 10% fetal bovine serum (FBS) and 5 mg/mL G418 (Gibco^®^, Thermo Fisher Scientific, Waltham, MA, USA, Cat# 10131027) at 1 × 10^7^ cells in a T75 flask. The flask was incubated at 37 °C in a carbon dioxide incubator until 90% confluence was reached for inoculation. Each flask was inoculated with 20 µL of virus stock [2]. One hour after incubation, the culture supernatant was removed, and cells were replenished with 10 mL of DMEM medium with 1% FBS, 100 U/mL penicillin-streptomycin, 100 U/mL nystatin, and 25 mM HEPES (Gibco^®^, ThermoFisher Scientific, Waltham, MA, USA, Cat# 15630130). The cells were incubated at 37 °C with 5% CO_2_ and observed daily for virus-induced cytopathic effect (CPE). Once cultures with more than 50% virus-induced CPE were expanded to large volumes in VeroE6/TMPRSS2 cells, the 50% tissue culture infective doses (TCID50) were determined in VeroE6/TMPRSS2 cells.

### 2.9. Live Virus Neutralizing Antibody Assay

A live virus neutralizing antibody assay using the SARS-CoV-2 wild-type (HKU-001a), BA.5.2, and XBB.1 strains was performed as previously described [14]. Briefly, serum samples were heat-inactivated at 56 °C for 30 min and were serially diluted in 2-folds with DMEM containing 1% FBS. Duplicates of each diluted serum sample were mixed with a SARS-CoV-2 virus isolate to reach a final concentration of 100 TCID_50_ and were incubated at 37 °C for 1 h. After incubation, 100 µL of the serum-virus mixture was added to VeroE6/TMPRSS2 cells that were seeded in 96-well plates 24 h before infection. The cells were incubated with the mixture at 37 °C. After incubation for 3 days, CPE was visually scored for each well by two independent observers. The 50% neutralization titer (NT_50_) was determined by using log (inhibitor) vs. normalized response-variable slope in GraphPad PRISM version 9.5.0. For statistical analysis, a value of 20 was assigned if the live virus-neutralizing antibody titer was <40. Similarly, a value of 2 was assigned if the live virus-neutralizing antibody titer was <4.

### 2.10. Determination of Viral Load by Quantitative Reverse Transcription-Polymerase Chain Reaction (qRT-PCR)

The experiments were performed as previously described [9]. Left lung and nasal turbinate tissues were homogenized and extracted for total RNA with the RNeasy Mini RNA Extraction Kit (Qiagen, Hilden, Germany, Cat# 74106). Nasal wash samples collected in 400 μL of viral transport medium were extracted for the total RNA using the QIAamp Viral RNA Mini Kit (Qiagen, Hilden, Germany, Cat# 52904). One-step qRT-PCR was performed for the detection of copies of the SARS-CoV-2 virus RdRp gene using a QuantiNova Probe RT-PCR Kit (Qiagen, Hilden, Germany, Cat# 208352) on a LightCycler 480 system (Roche). Primers and probes were listed as follows: SARS-CoV-2 RdRp, Forward: 5′ CGCATACAGTCTTRCAGGCT 3′; Reverse: 5′ GTGTGATGTTGAWATGACATGGTC 3′; Probe (5′ to 3′): FAM-TTAAGATGTGGTGCTTGCATACGTAGAC-lABkFQ. β-actin, Forward: 5′ ATGGCCAGGTCATCACCATTG 3′; Reverse: 5′ CAGGAAGGAAGGCTGGAAAAG 3′; Probe (from 5′ to 3′): Cy5-AGCGGTTCCGTTGCCCTGAG-IABkFQ.

### 2.11. Statistical Analysis

Statistical analysis was performed using PRISM 9.5.0. NT_50_ against wild-type, BA.5.2, and XBB.1 strains and compared using one-way ANOVA and corrected for multiple comparisons using Dunn’s multiple comparisons test. *p* < 0.05 was considered statistically significant.

## 3. Results

### 3.1. Construction of Expression Vector

RBD, S1, and S gene fragments of SARS-CoV-2 were cloned into pFastbac dual followed by transformation to DH10bac for recombinant bacmid DNA generated using the Bac-to-Bac system [18]. An N-terminal included 41 amino acids of the gp67 signal peptide, and a C-terminal 6xHis tag was included for secretion and purification purposes. A C-terminal T4 fibritin trimerization motif and a flexible linker were initially used for recombinant trimeric protein folding (Figure 1).

### 3.2. Expression and Purification of Recombinant Protein

Four recombinant His-tag trimeric spike RBDs of wild-type SARS-CoV-2 were expressed in EXPI SF9 cell lines, which were soluble in culture supernatant and pure after purification through a size exchange column. Their sizes are 36.69 kDa (trimeric RBD), 84.59 kDa (trimeric S1), 61.79 kDa (Fc-RBD), and 142.1 kDa (trimeric full-length spike) (Supplementary Figure S1).

### 3.3. Humoral Immune Response in Balb/c Mice

Blood collected on day 42 after the last dose of vaccination showed that two doses of intramuscular CoronaVac followed by one intranasal dose of boosting by trimeric spike RBD (Figure 2A) had significantly higher total IgG for RBD than three doses of intramuscular CoronaVac (Figure 2B). Moreover, two doses of intramuscular CoronaVac followed by one dose of intranasal Fc-RBD had significantly higher IgG1 than three doses of intramuscular CoronaVac (Figure 2C). No significant differences in serum IgG2a levels were found (Figure 2D). Notably, one dose of intranasal boosting by all three types of recombinant proteins (trimeric RBD, trimeric Fc-RBD, or trimeric S) elicited a significantly higher total IgG in BAL samples than three doses of intramuscular CoronaVac (Figure 2E). Rather unexpectedly, one intranasal boost with trimeric spike S1 did not boost IgG and IgA significantly in BAL samples (Figure 2F).

As for neutralizing antibody titres, none of the boosting approaches produced a significant difference against wild-type SARS-CoV-2 (Figure 2G). However, a single intranasal boosting with Fc-RBD induced significantly higher neutralizing antibody titres against the Omicron BA.5.2 subvariant (52.78, 95% CI: 19.78–140.8 for RBD intranasal boosting, 320.0, 95% CI: 174.1–588.1 for Fc-RBD intranasal boosting; 45.95, 95% CI: 14.96–141.1 for S1 intranasal boosting; 80, 95% CI: 23.69–270.2 for S intranasal boosting; and 45.95 95% CI: 22.36–94.4 for three intramuscular doses of CoronaVac) (Figure 2H). We therefore selected the recombinant Fc-RBD for further study.

### 3.4. Trimeric Fc-RBD Vaccine Booster Protects K18-hACE2 Mice against SARS-CoV-2 BA.5.2 Variant

After priming with two intramuscular doses of CoronaVac, a single dose of intranasal boosting with Fc-RBD was compared with the control group’s three doses of intramuscular CoronaVac (Figure 4A). In terms of the adaptive humoral immune response just before virus challenge, a significantly higher neutralizing antibody titer against both wild-type SARS-CoV-2 (1689, 95% CI: 782.2–3647) and the Omicron BA.5.2 subvariant (242.5, 95% CI: 76.43–769.5) were found in the group boosted by a single intranasal dose of Fc-RBD, but not in the group boosted by a single intramuscular dose of Fc-RBD or the group with three intramuscular doses of CoronaVac (Figure 4B,C). While the respiratory tract viral loads of the mice that received three intramuscular doses of CoronaVac and those that received PBS were similar at day four post-infection, the former vaccination strategy did protect the mice from dying from day five post-infection onwards, as the vaccination might have reduced immunopathologies in the mice. As expected, the viral load in the nasal turbinates of the group boosted by a single intranasal dose of Fc-RBD was significantly lower than that of the other groups, although the effect was less obvious in the lung tissues (Figure 4D,E). Upon challenge with the Omicron BA.5.2 subvariant, a 20% weight loss was found on day seven post-challenge, with only a 40% survival on day six post-challenge in unvaccinated mice, while no mortality or significant weight loss was observed in vaccinated mice (Figure 4F,G).

### 3.5. Immune Response after Boosting with Two Doses of Intranasal Trimeric Fc-RBD against Omicron Subvariants BA.5.2 and XBB.1

**Figure 5:**
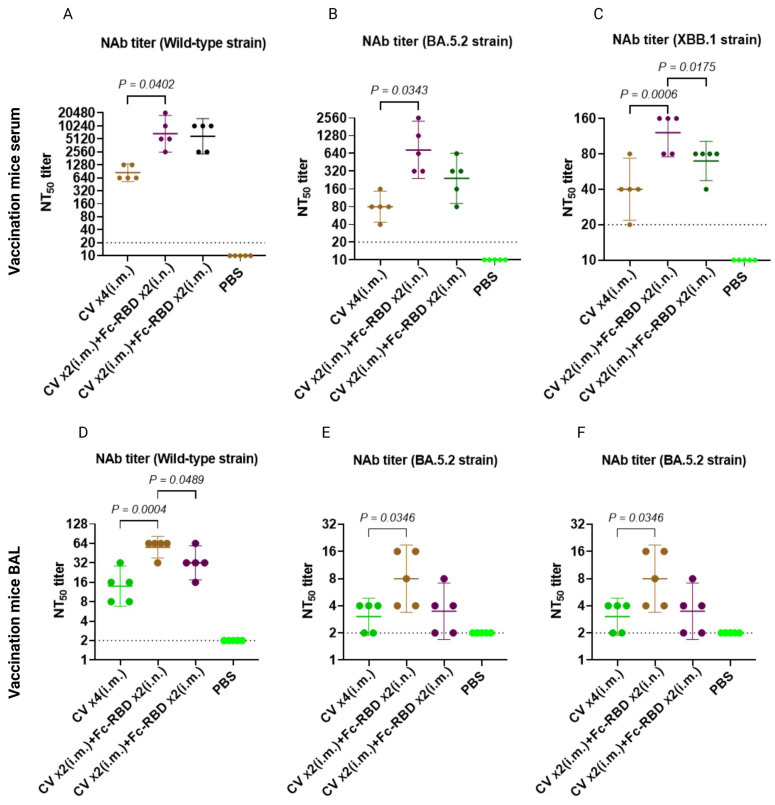
Comparison of humoral immune responses between two intramuscular doses of CoronaVac plus two doses of intranasal Fc-RBD or two doses of intramuscular Fc-vaccine booster with four intramuscular doses of CoronaVac in Balb/c mice. (**A**) Wild-type SARS-CoV-2; (**B**) Omicron BA.5.2 subvariant; and (**C**) Omicron XBB.1 subvariant neutralization of pooled sera collected on day 56 was quantitated by conventional live virus neutralization tests (cVNT). Bronchoalveolar lavage neutralizing antibody titers from samples collected on day 56 were quantitated by cVNT for (**D**) wild-type SARS-CoV-2; (**E**) Omicron BA.5.2 subvariant; and (**F**) Omicron XBB.1 subvariant. A one-way ANOVA test was used for statistical analysis. Abbreviations: CV, CoronaVac; i.m., intramuscular; and i.n., intranasal.

To see if boosting with two doses of intranasal Fc-RBD or intramuscular CoronaVac would make a difference in terms of humoral immune response, we examined the serum and BAL antibody response two weeks after the last dose of boosting (day 56) (Figure 3). Two boosting doses of intranasal Fc-RBD induced significantly higher serum neutralizing antibodies against both wild-type SARS-CoV-2 and Omicron subvariants BA.5.2 and XBB.1. Notably, Fc-RBD boosted a robustly induced geometric mean microneutralization antibody titer (GMT) against the Omicron BA.5.2 subvariant (735.2, 95% CI: 239.4–2258) but only modestly against XBB.1 (121.3, 95% CI: 75.68–194.3), which suggested that XBB.1 is more immune evasive than BA.5.2 (Figure 5A–C). A similar trend for a neutralizing antibody titre in BAL was found at a lower titre (Figure 5D–F).

## 4. Discussion

In this study, we used recombinant DNA technology to fuse the biologically active RBD of the SARS-CoV-2 spike protein with the Fc fragment of mouse immunoglobulin IgG1 to produce our recombinant protein vaccine. The RBD peptide targets most neutralizing antibodies against SARS-CoV-2 [23,24]. Our recombinant protein vaccine retains the immunogenicity of RBD, and the Fc fragment facilitates its binding to and uptake by antigen-presenting cells (APCs), which may reduce the degradation of the free vaccine by proteases in the body, thereby increasing the half-life of vaccines [25,26,27]. Moreover, the interaction between the Fc fragment and APCs enhances the immunogenicity of the vaccine by inducing a longer-lasting immune activation against the RBD [26,27]. During subsequent doses of booster vaccination when serum antibodies against the RBD are present, the Fc fragment may also add more vaccine uptake by APCs in addition to the normal uptake of the immune complex by Fc receptors for IgG (FcγR) mediated antigen uptake of dendritic cells, which present antigens to activate CD4+ and CD8+ T cells [28]. Liu and colleagues have recently shown that an RBD-Fc-based vaccine can effectively induce highly potent neutralizing antibody responses against coronavirus infections, such as SARS-CoV-2, SARS-CoV, and bat SARSr-CoV-WIV1. Furthermore, the RBD-Fc-based vaccine can protect hACE2-Tg mice from the SARS-CoV-2 challenge [29]. Steers and colleagues have also demonstrated that the Fc fragment fusion vaccine of HIV-1 Gag p24 can improve mice’s antigen-specific humoral immune response [30]. Our study shows that intranasal boosting with the Fc-RBD vaccine induced significantly higher neutralizing antibody titres against the Omicron BA.5.2 subvariant (NT50: 320 vs. 52.78) and protection of K18-hACE2 mice from the BA.5.2 virus challenge.

Here, we demonstrated that one or two intranasal boosters with Fc-linked trimeric spike RBD from wild-type SARS-CoV-2 can induce a good neutralizing antibody response against wild-type SARS-CoV-2 and Omicron subvariants, including BA.5.2 and XBB.1, in the serum and at a lower titre in the BAL of only intramuscularly vaccinated mice. The neutralizing antibody titers of this approach using intramuscular whole virion priming and intranasal boosting with Fc-RBD using vaccines containing the same wild-type SARS-CoV-2 spike RBD are significantly higher than those found in mice vaccinated with four intramuscular doses of inactivated whole virion vaccine. These intranasally vaccinated mice also had significantly lower viral loads in the nasal turbinates, suggesting better protection of the upper airway, which is the predilected site of infection by Omicron subvariants.

Of the four widely available COVID-19 vaccines using inactivated whole virions, mRNA, an adenoviral vector, and recombinant spike protein, the inactivated whole virions vaccine is the most widely used in developing countries because of its safety and effectiveness for the prevention of severe COVID-19 [2]. However, none of these injection vaccines using wild-type SARS-CoV-2 or spike were able to induce a good neutralizing antibody response and cross-protection against Omicron subvariants, especially in the upper respiratory tract where Omicron predominantly infects rather than the lung, which is more commonly involved in infection caused by wild-type SARS-CoV-2 [31].

While a neutralizing antibody is largely responsible for prevention of SARS-CoV-2 infection, recovery from an established infection is likely mediated by both cytotoxic CD8 T lymphocytes to clear virus-infected cells and CD4 lymphocytes-mediated neutralizing antibody response [32]. Although an inactivated whole virion vaccine can induce neutralizing antibodies and some degree of T cell response, the level is modest, which could be due to spike protein denaturation during inactivation, the use of the weaker alum adjuvant, and the large amount of competing non-neutralizing immunogens from viral nucleocapsid, membrane, and envelope proteins. In terms of serum neutralizing antibody response, the mRNA vaccine consistently induced an almost 10-fold higher titre than that of the inactivated vaccine [33]. The adenoviral-vectored vaccine also has a large amount of non-neutralizing immunogens from the adenoviral vector. Even the very potent mRNA vaccine and the recombinant spike protein vaccine with potent saponin have many non-neutralizing antigenic epitopes that may prime the recipient to produce non-neutralizing antibodies with repeated boosting. Though mRNA, adenoviral-vectored, and saponin-adjuvanted recombinant spike vaccines can induce better cell-mediated immunity than inactivated whole virion vaccines, no standardized assay for T cell response or threshold value for a specific T cell response have been clearly linked to protection in large field trials of COVID-19 vaccination. Neutralizing antibody titre remains the only useful immune correlate of protection and was therefore used in this study.

Many strategies have been used to improve the immunogenicity by the induction of neutralizing antibodies. Most have used a potent adjuvant, which may induce just as many non-neutralizing antibodies and the antigenic sin from priming. Instead of the whole spike protein, some just used the spike RBD to minimize the amount of non-neutralizing antigenic epitope and the associated antigenic sin. Others tried to change the amino acid residues around the RBD to improve immunogenicity by using the RBD (F318-C617) [34], RBD (R319-F541) [35], RBD (N331-V524) [36,37,38,39], RBD (P330-E583) [40], RBD (R319-F541) [41], and RBD (Arg319-N532) [29] protein as the immunogen. Others added glycosylation sites around the RBD to shield the non-neutralizing epitopes of the RBD [42]. In this study, we used a Fc-linked trimeric spike RBD to improve the uptake and presentation of the trimeric spike RBD, and we used intranasal administration to achieve a more robust immunity in the upper airway. Twelve studies [29,34,35,36,37,38,39,40,41,43,44,45] have tried to use Fc-RBD subcutaneously or intramuscularly with mostly alum, one Freund’s, one MF59, manganese, or Montanide TM ISA720 adjuvants in mice, and one non-human primate model; but only one study has used intranasal vaccination in Balb/c mice, and it did not try to use it for boosting after priming with intramuscular inactivated vaccine.

## 5. Conclusions

In summary, priming by using two doses of intramuscularly inactivated vaccine followed by intranasal boosting with Fc-RBD can induce a good neutralizing antibody response against wild-type SARS-CoV-2 and the most recent Omicron BA.5.2 and XBB.1 subvariants, as well as provide upper airway protection with reduced viral load in mice.

## Data Availability

All data can be found in this manuscript.

## Supplementary Materials

The following supporting information can be downloaded at https://www.mdpi.com/article/10.3390/v15030687/s1, Figure S1: Representative size exclusion chromatography profile and Sodium dodecyl sulfate-polyacrylamide gel electrophoresis (SDS-PAGE) analysis of different recombinant protein using an ENrichTM SEC 650 column in 1xPBS.
