# Supplementary material for: Intranasal Boosting with Spike Fc-RBD of Wild-Type SARS-CoV-2 Induces Neutralizing Antibodies against Omicron Subvariants and Reduces Viral Load in the Nasal Turbinate of Mice
Source: Viruses. 2023 Mar 6;15(3):687. doi: 10.3390/v15030687 (PMC10052291; doi:10.3390/v15030687)
Supplement: Supplementary file 1 [file viruses-15-00687-s001.zip › viruses-2216350-supplementary.pptx]

## Slide 1
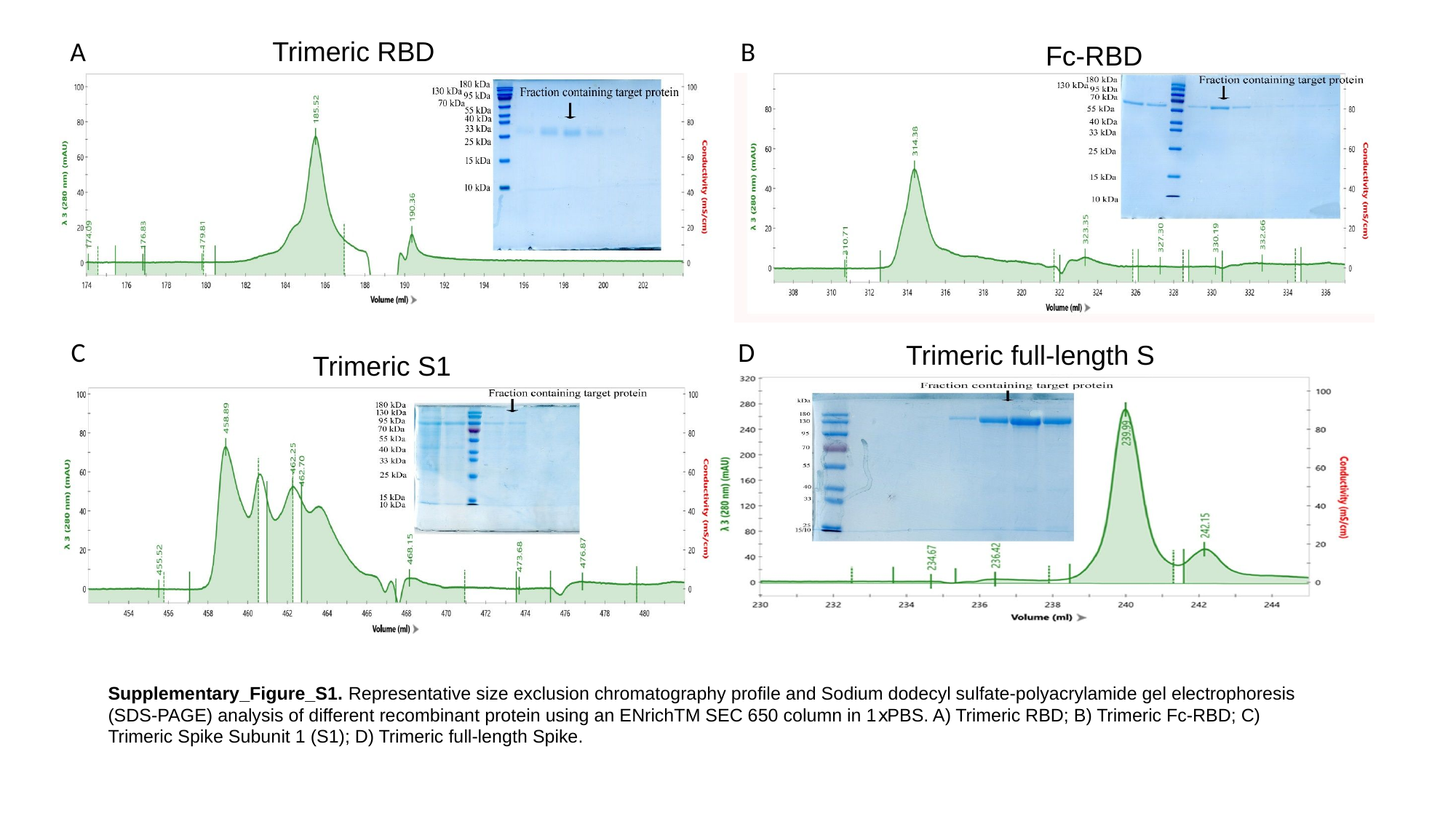

A
Trimeric RBD
B
Fc-RBD
C
D
Trimeric full-length S
Trimeric S1
Supplementary_Figure_S1. Representative size exclusion chromatography profile and Sodium dodecyl sulfate-polyacrylamide gel electrophoresis (SDS-PAGE) analysis of different recombinant protein using an ENrichTM SEC 650 column in 1ⅹPBS. A) Trimeric RBD; B) Trimeric Fc-RBD; C) Trimeric Spike Subunit 1 (S1); D) Trimeric full-length Spike.
